# Metabolic mapping by use of high-resolution magic angle spinning ^1^H MR spectroscopy for assessment of apoptosis in cervical carcinomas

**DOI:** 10.1186/1471-2407-7-11

**Published:** 2007-01-17

**Authors:** Heidi Lyng, Beathe Sitter, Tone F Bathen, Line R Jensen, Kolbein Sundfør, Gunnar B Kristensen, Ingrid S Gribbestad

**Affiliations:** 1 Department of Radiation Biology, Rikshospitalet-Radiumhospitalet Medical Center, Oslo, Norway; 2 Department of Circulation and Medical Imaging, Norwegian University of Science and Technology, Trondheim, Norway; 3 Department of Gynecologic Oncology, Rikshospitalet-Radiumhospitalet Medical Center, Oslo, Norway; 4 Department of Medical Informatics, University of Oslo, Oslo, Norway

## Abstract

**Background:**

High-resolution magic angle proton magnetic resonance spectroscopy (HR ^1^H MAS MRS) provides a broad metabolic mapping of intact tumor samples and allows for microscopy investigations of the samples after spectra acquisition. Experimental studies have suggested that the method can be used for detection of apoptosis, but this has not been investigated in a clinical setting so far. We have explored this hypothesis in cervical cancers by searching for metabolites associated with apoptosis that were not influenced by other histopathological parameters like tumor load and tumor cell density.

**Methods:**

Biopsies (n = 44) taken before and during radiotherapy in 23 patients were subjected to HR MAS MRS. A standard pulse-acquire spectrum provided information about lipids, and a spin-echo spectrum enabled detection of non-lipid metabolites in the lipid region of the spectra. Apoptotic cell density, tumor cell fraction, and tumor cell density were determined by histopathological analysis after spectra acquisition.

**Results:**

The apoptotic cell density correlated with the standard pulse-acquire spectra (p < 0.001), but not with the spin-echo spectra, showing that the lipid metabolites were most important. The combined information of all lipids contributed to the correlation, with a major contribution from the ratio of fatty acid -CH_2 _to CH_3 _(p = 0.02). In contrast, the spin-echo spectra contained the main information on tumor cell fraction and tumor cell density (*p *< 0.001), for which cholines, creatine, taurine, glucose, and lactate were most important. Significant correlations were found between tumor cell fraction and glucose concentration (*p *= 0.001) and between tumor cell density and glycerophosphocholine (GPC) concentration (*p *= 0.024) and ratio of GPC to choline (*p *< 0.001).

**Conclusion:**

Our findings indicate that the apoptotic activity of cervical cancers can be assessed from the lipid metabolites in HR MAS MR spectra and that the HR MAS data may reveal novel information on the metabolic changes characteristic of apoptosis. These changes differed from those associated with tumor load and tumor cell density, suggesting an application of the method to explore the role of apoptosis in the course of the disease.

## Background

Apoptosis plays an important role in cancer development, progression, and response to therapy [1,2]. Malignant cells have defects in cellular death pathways and therefore the ability to circumvent apoptosis and survive under abnormal conditions [3]. Low apoptotic activity has been associated with aggressiveness and treatment resistance of many tumor types, including cervical carcinomas [4,5]. Apoptosis is also an important mode of cell death after chemo- and radiotherapy, although its relative contribution to the overall cell death has not been clarified [2]. Information on the apoptotic activity in tumors and the metabolic changes involved may give valuable insight into the mechanisms underlying cancer progression and treatment response. Considerable effort is therefore put into the development of clinically useful methods for detection and exploration of apoptosis.

MR spectroscopy allows for a broad metabolic mapping of tissues at the molecular level that may be used to investigate the balance between cell death and survival [6]. Detailed biochemical characterization of hundreds of metabolites is provided in a single experiment, yielding specific information on the turnover of lipids, carbohydrates, and polypeptides [7]. Experimental studies, both *in vivo *and based on tissue extracts, have indicated a potential of the method to detect apoptosis from the levels of cholines and fatty acids [8,9]. Recent developments by utilization of the HR MAS technique have enabled production of high quality spectra from intact tissues without the artefacts introduced by extraction procedures [10]. Histological studies can be performed on the samples after spectrum acquisition, yielding information on the underlying causes of the metabolic data.

There is currently a large interest in exploring the clinical potential of HR MAS MRS. Several studies have shown that malignant lesions can be distinguished from benign ones in the spectra [11-16], and utilizations of the method for prediction of tumor aggressiveness and treatment outcome have been proposed [12,14,15]. Only a few HR MAS studies have investigated the relationship between apoptosis and spectral data [17-20]. The studies are based on rat gliomas with high apoptotic activity and homogeneous tissue composition compared to human tumors. Normal cells within the tumor contribute significantly to the MR signals and increase the complexity of clinical data. The proportion of malignant cells and their apoptotic activity may be interrelated, necessitating identification of spectral characteristics of apoptosis that differ from those associated with tumor load and tumor cell density. The clinical relevance of the previous findings is therefore not clear.

The present work was conducted to explore the utilization of HR MAS MRS for detection of apoptosis in cervical cancers. Spectral characteristics specific for apoptotic cell density, tumor cell fraction, and tumor cell density, as determined by histopathologic analysis of the samples after spectrum acquisition, were searched for. Biopsies taken before the start of treatment and after one week of therapy were included to achieve a broad, but clinically relevant, range of the histopathologic parameters. Significant relationships between the HR MAS MR spectra and the histopathology were found and metabolites specific for apoptosis that were clearly distinguished from those associated with tumor cell fraction and tumor cell density were identified.

## Methods

### Patients and tumor samples

Twenty-three patients with primary squamous cell carcinoma of the uterine cervix were included. Tumor stage (The Fédération Internationale des Gynaecologistes et Obstetristes) was 1b (1 patient), 2b (16), and 3b (6). All patients were treated with curative intent. Radiotherapy was given to all but one patient, who received surgery. External irradiation, a total of 50 Gy to tumor and parametria and 45 Gy to the rest of the pelvic region, was given in fractions of 2 and 1.8 Gy, respectively. Brachytherapy, 21 Gy in five fractions, was thereafter employed. Nineteen patients received adjuvant cisplatin (40 mg/m^2^) weekly during the period of external radiation, starting the same day as the radiotherapy. The study was approved by the local ethical committee, and written informed-consent was obtained from all patients.

Tumor biopsies, 5 × 5 × 5 mm in size, were taken before the start of treatment from all twenty-three patients and after 1 week (10 Gy) of external radiotherapy from twenty patients. Two pre-treatment biopsies were included for one of the patients, leading to a total of 44 samples. Efforts were made to select samples from the viable tumor tissue, avoiding necrotic regions that were sometimes visible particularly after 1 week of therapy. The biopsies were immediately frozen in liquid nitrogen and stored at -80°C until MR analysis.

### MR experiments

The samples were cut to fit a MAS rotor (50 μL, mean sample weight 16.2 mg), and added phosphate buffered saline made up in D_2_O with trimethylsilyl propionic acid (TSP, 1.4 mM) as a chemical shift reference and standard for quantification of metabolites. HR MAS experiments were performed on a Bruker Avance DRX600 spectrometer equipped with a ^1^H/^13^C MAS probe with gradient aligned with the magic angle axis (Bruker BioSpin GmbH, Germany), as reported earlier [14]. Samples were spun at 5 kHz at an instrumental temperature setting of 4°C. Two spectra were recorded for each sample. A standard pulse-acquire spectrum with suppression of water signal (zgpr; BRUKER) allowed for quantification of metabolite concentrations and provided information on lipid metabolites, whereas a spin-echo spectrum with suppression of both water and lipid signal (cpmgpr; BRUKER) enabled detection of non-lipid metabolites in the spectral region of the lipids. The standard pulse-acquire experiments included a 60° flip angle over a spectral region of 20.0 ppm after 3.0 sec of water presaturation. The free induction decay (FID) was acquired into 64 K points during 2.72 sec, and 32 transients were collected. The spin-echo experiments were performed using 2 sec of water suppression prior to a 90 degrees excitation pulse. A total of 128 transients over a spectral region of 16.7 ppm were collected into 32 K points during 1.64 sec. The T_2_-filtering was obtained using a delay of 1 ms repeated 136 times, resulting in 285 ms effective echo time. The repetition time was 3.93 sec. Spectral assignments were performed based on our previous HR MAS study of cervical cancers [13]. The samples were fixed in formalin after the MR experiment. A time period of 30 min was used for spectrum acquisition, and the mean period from the start of the experiment to fixation was 1 hour 55 min. The FIDs were Fourier transformed after 0.3 Hz exponential line broadening, and the phase was manually corrected. A linear baseline correction was applied. Chemical shifts were referenced to the TSP peak at 0 ppm.

### Histopathologic examinations

The samples were embedded in paraffin casts. A 5 μm thick section from the middle part of each biopsy was subjected to histological analysis for determination of apoptotic cell density, tumor cell fraction, and tumor cell density, as described previously [21], assuming that the data of this section were representative for the entire biopsy. One biopsy contained solely necrosis. Stroma and tumor cells, but no necrosis, were seen in the others. Since necrosis most often affects many adjacent cells at the same time and is easily visible in the microscope, the proportion of necrosis was assumed to be zero in the latter biopsies. In contrast, apoptosis may often occur in single cells spread throughout the tissue.

To detect the apoptotic cells, the sections were stained by use of the Apotag *in situ *apoptosis detection kit (Oncor, Gaithersburg, MD), which is based on the terminal deoxynucleotidyl transferase (TdT)-mediated dUTP-biotin nick end labeling (TUNEL) method. Slide preparation and staining were performed as described by the manufacturer. A biopsy from a neoplastic lymph node of a patient with B-cell non-Hodgkin's lymphoma served as a positive control, whereas negative controls received no TdT. Apoptotic cell density was defined as the number of apoptotic tumor cells per mm^2 ^of tissue (including stroma and tumor tissue), and was calculated as:

*A*_*t *_= *F*_*c*_·*A*_*c *_    (1)

where *A*_*t *_is apoptotic cell density, *F*_*c *_is tumor cell fraction, and *A*_*c *_is number of apoptotic nuclei per mm^2 ^of tumor tissue. *F*_*c *_was determined in hematoxylin and eosin (HE) stained sections by point counting at a magnification of 100×, and *A*_*c *_was determined by counting all apoptotic cells within the tumor tissue at high magnification (400×).

Tumor cell density, defined as number of tumor cell nuclei per mm^2 ^of tissue (including stroma and tumor tissue), was calculated as:

*D*_*t *_= *F*_*c*_·*D*_*c *_   (2)

where *D*_*t *_is tumor cell density, *F*_*c *_is tumor cell fraction, and *D*_*c *_is number of nuclei per mm^2 ^of tumor tissue. *D*_*c *_was determined in HE stained sections by counting all nuclei within selected fields in the tumor tissue at high magnification (400×).

Apoptotic cell density, tumor cell fraction and tumor cell density were also determined in nine samples that had not been subjected to MR examinations. These data were compared to the data of the HR MAS samples, to search for systematic changes in the histopathology caused by the MR experiement. The samples were prepared and analysed as described above.

### Partial least square regression (PLS)

The MR spectra and histopathological parameters were analysed by PLS to identify the metabolites that were most strongly related to apoptotic cell density, tumor cell fraction, and tumor cell density. The analysis relates variations in one or several dependent variables; *i.e*, the histopathological parameters, to the variations of several independent variables; *i.e*., the spectral data, with explanatory or predictive purposes. The method performs well when the independent variables are collinear, which can be expected with MR data. The information in the original MR spectra was projected onto a number of principal components (PCs). The histolopathological parameters were actively used in estimating the PCs, ensuring that the first PCs were those that were most relevant for predicting the parameter.

The MR spectra were converted to ASCII-format, peak aligned according to a recently reported algorithm [22,23], and imported into the Unscrambler package (CAMO process AS, Norway) for PLS analysis. The region between 0.7 and 2.2 ppm, mainly containing broad resonances from lipids with signals from the fatty acid protons - CH_3 _(0.9 ppm), - (CH_2_)n (1.3 ppm), - CH_2 _- CH_3 _(1.35 ppm), - CH_2 _- CH_2 _- CO (1.58 ppm), - CH = CH - CH_2 _- CH_2 _(2.02 ppm), was used in the standard spectra. The ethanol triplet at 1.19 ppm that occurred due to the laboratory sterilizing procedure, was excluded. In the spin-echo spectra the regions containing the peaks for glucose, lactate, *myo*-inositol, taurine, glycerophosphocholine (GPC), phosphocholine (PC) choline, and creatine were selected for further analysis. Baseline offset was adjusted, and the spectra were scaled by mean normalisation; *i.e*., the area below the curve was made equal for all spectra. PLS calibration of apoptotic cell density, tumor cell fraction, and tumor cell density to the spectral variables was performed in separate runs. The model calibrations were performed with mean centered data; *i.e*., the average of each variable subtracted from each of the data values in the variable. This centering means that the results can be interpreted in terms of deviations from the average. Full cross-validation (leave-one-out) was applied in model calibration. The number of PCs to retain in the model was determined by finding the principal component where total residual y-variance and root mean square error of prediction were minimised. Loading profiles of the principal components were generated to visualize the relative contribution of the metabolites to the model. Pearson correlation analysis was used to find the correlation between the predicted and measured parameter.

### Estimation of metabolite concentrations

To further explore the association between metabolites and histopathology, correlation analyses based on individual metabolite concentrations and metabolite ratios were performed. The concentrations were calculated for β-glucose, GPC, PC, choline, creatine, lactate, fatty acid -CH_2 _(1.3 ppm), fatty acid -CH_3 _(0.9 ppm), and TSP from the standard pulse-acquire spectra, as described previously [14]. The spectral regions 4.6 to 4.7 ppm (β-glucose), 3.4 to 2.9 ppm (GPC, PC, choline, and creatine), 1.8 to 0.5 ppm (lipids and lactate), and -0.1 to 0.1 ppm (TSP) were individually baseline corrected, using a linear function. Peak areas were calculated using combined Lorentzian and Gaussian line functions (Voigt area) in a curve-fitting program (PeakFit, Systat Software Inc., Richmond, CA). The correlation coefficient, *r*, of the fit was 0.95 or larger for all area calculations. The peak area of the metabolite relative to the area of the internal standard TSP and sample wet weight was used as a measure of metabolite concentration. The concentrations were related to the histopathological parameters by use of Pearson correlation analysis and the SPSS software.

## Results

### Histopathology and spectral characteristics

**Figure 1:**
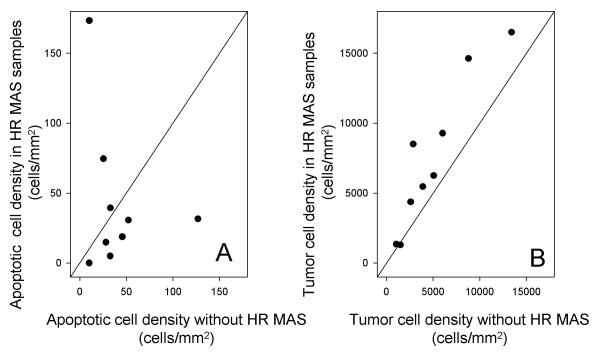
Apoptotic cell density (A) and tumor cell density (B) in cervical cancer biopsies after HR MAS MR spectroscopy *versus *the corresponding data of samples from the same tumors but not subjected to MR spectroscopy. MR spectroscopy was performed at an instrumental setting of 4°C. The HR MAS samples were fixed in formalin after spectrum acquisition, using an average time period of 1 hour 55 min from the start of the experiment to fixation. Each point represents the data of a single biopsy. The values were calculated as number of apoptotic tumor cells (A) and tumor cells (B) per mm^2 ^of tumor tissue. Lines of unity are shown. Note that there was no increase in apoptotic cell density or decrease in tumor cell density caused by the MR experiment. The increased tumor cell density (B) was probably due to a minor increase in the thickness of the histological sections from the HR MAS samples as compared to the others.

The histolopathology differed considerably among the samples. To avoid confounding effects due to the influence of necrosis on the spectra, the necrotic sample was not included in the statistical analyses but was handled separately. Apoptotic cell density, tumor cell fraction, and tumor cell density of the other samples ranged from 0 – 189 apoptotic cells per mm^2 ^(median 16 apoptotic cells per mm^2^), 8 – 100% (median 51%), and 214 – 11542 cells per mm^2 ^(median 2357 cells per mm^2^), respectively. Apoptotic cell density was generally higher whereas tumor cell fraction and tumor cell density were lower in the biopsies taken during therapy compared to the pretreatment ones from the same tumor, reflecting treatment induced apoptosis and cell death. However, when biopsies from different tumors were compared, no such trend was observed. Hence, many of the pretreatment biopsies had more apoptosis and lower tumor cell fraction and tumor cell density than biopsies taken during therapy from other tumors. The morphology of the samples did not change during the MR experiments, and there was no increase in apoptotic cell density or decrease in tumor cell density either (Figure 1).

**Figure 2:**
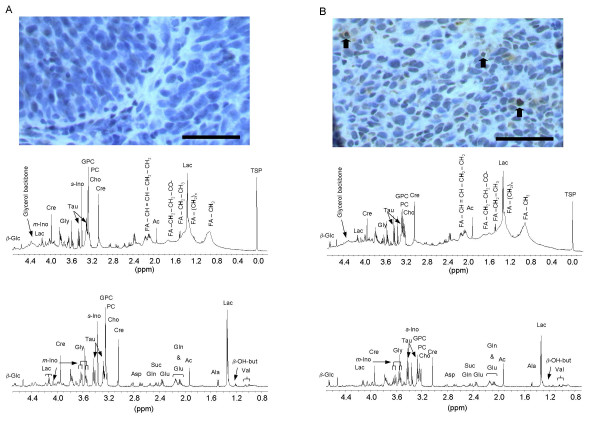
Histological section and HR MAS MR standard pulse-acquire spectrum (upper) and spin-echo spectrum (lower) of two cervical cancer biopsies, one with no apoptosis (A) and another with significant apoptotic activity (B). The histological sections were stained for apoptotic cells by use of the TUNEL method after spectrum acquisition. The biopsy presented in (A) had a tumor cell fraction of 85%, tumor cell density of 9552 cells/mm^2^, and no apoptosis, whereas the corresponding data of the biopsy in (B) were 35%, 3606 cells/mm^2^, and 8.3 apoptotic cells/mm^2^. Bars in the histological sections represent 50 μm, and arrows in (B) point to apoptotic cells. Note the highly preserved morphology of the samples after spectrum acquisition. Spectral assignments are abbreviated: β-Glc, β-glucose; Lac, lactate; *m*-Ino, *myo*-inositol; Cre, creatine; Gly, glycine; Tau, taurine; *s*-Ino, *scyllo*-inositol; GPC, glycerophosphocholine; PC, phosphocholine; Cho, choline; FA, fatty acids; TSP, trimethylsilyl propionic acid; Asp, aspartate; Gln, glutamine; Suc, succinate; Glu, glutamate; Ac, acetate; Ala, alanine; β-OH-but, β-hydroxybutyrate; Val, valine. The position of hydrogens in fatty acids giving rise to the different peaks is marked in bold after the notation FA in the upper spectra.

All MR spectra were highly resolved and comparable to our previous results for cervical cancers [13]. Broad resonances from lipids and macromolecules, as well as sharp peaks from smaller compounds, like lactate, amino acids, and cholines, could be seen in the standard pulse-acquire spectra, whereas amino acids, like alanine and valine, were detected in the lipid region of the spin-echo spectra in addition to the other compounds (Figure 2). Pronounced differences were often observed between spectra acquired from different biopsies, especially in the region above 3.0 ppm, as demonstrated in Figures 2A and 2B. The differences could be seen regardless of whether the pre-treatment biopsies or all samples were compared. They were therefore not a general effect of the treatment, but reflected biological characteristics of the tissues.

### Relationships between histopathology and spectral profiles

**Figure 3:**
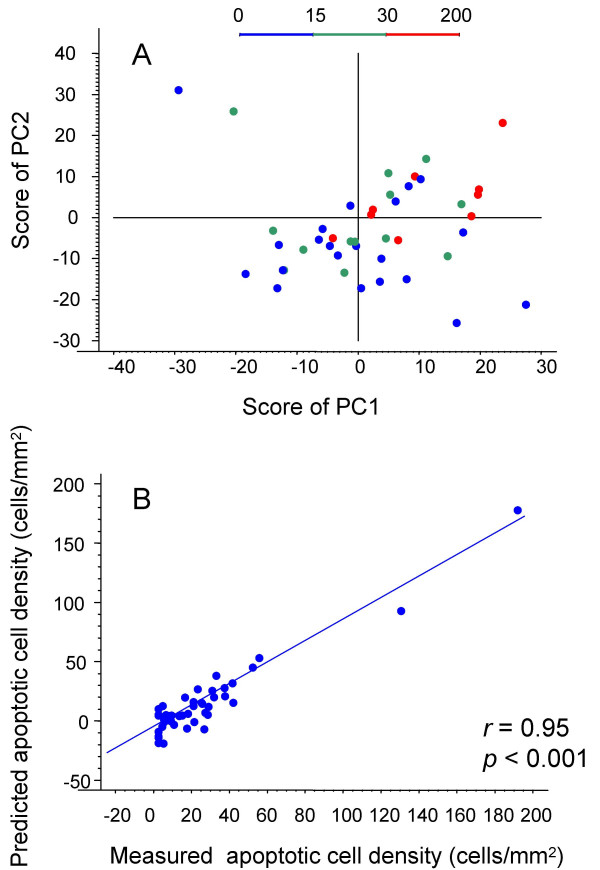
Score plot of first principal component (PC1) *versus *second principal component (PC2) from partial least square (PLS) regression calibration of apoptotic cell density to single-pulse spectral data (A), and the predicted *versus *measured apoptotic cell density (B). Each point represents the data of a single biopsy. In (A) the color code for apoptotic cell densities (cells/mm^2^) is shown. The Pearson correlation coefficient and *p*-value are marked in (B). Total residual y-variance and root mean square error of prediction were minimised by retaining 11 PCs in the model. These 11 PCs accounted for 98% of the total x-variation, and 92% of the total y-variation. An informative loading profile could not be generated due to the high number of principal components that were retained in the model.

Apoptotic cell density showed a significant correlation to the standard pulse-acquire spectra in PLS analysis (Figure 3). No association was found between the spin-echo spectra and apoptotic cell density, suggesting that the lipid region contained the main information on apoptosis. The PLS model had eleven valid principal components, and a single informative loading profile could therefore not be generated. However, the highly apoptotic samples had generally a high score of PC1 compared to the others (Figure 3A). A strong correlation was found between the predicted and measured apoptotic cell density (*r *= 0.95, *p *< 0.001) (Figure 3B), indicating that the apoptotic cell density could be well assessed from the spectral data. The high correlation coefficient was partly caused by two highly apoptotic samples. However, the relationship was significant even when these samples were excluded (*r *= 0.53, *p *< 0.001), showing that the result did not rely on them alone.

**Figure 4:**
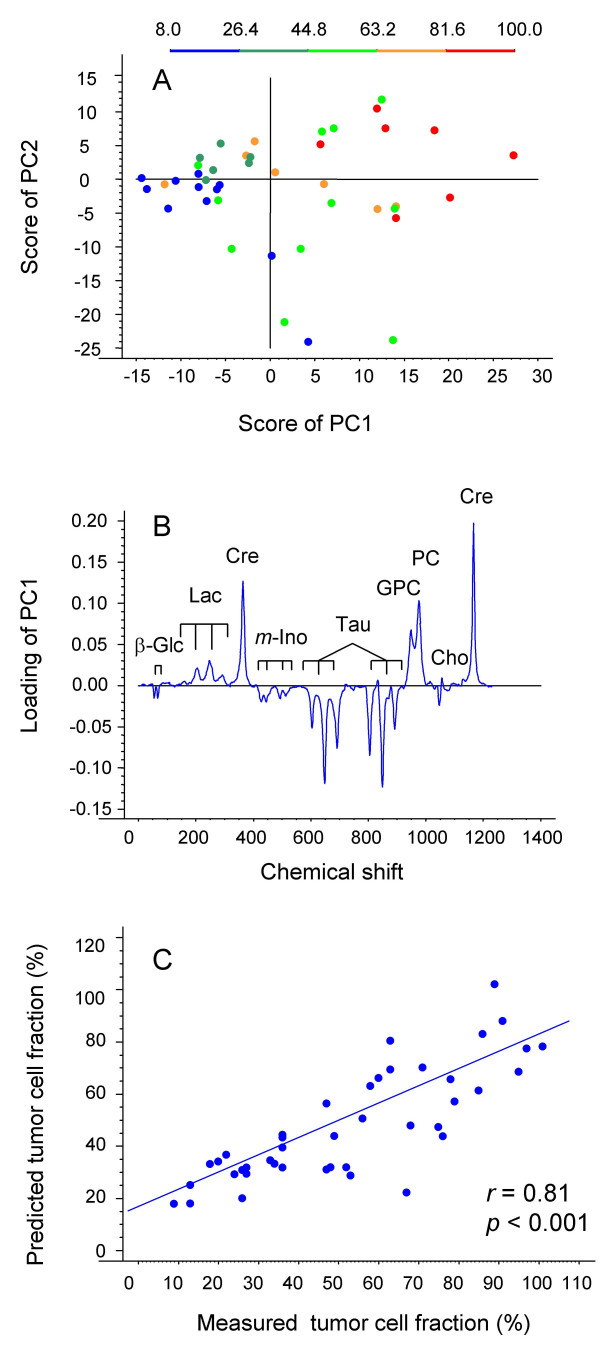
Score plot of first principal component (PC1) *versus *second principal component (PC2) from partial least square (PLS) regression analysis of tumor cell fraction to spin-echo spectral data (A), the corresponding loading profile of PC1 (B), and the predicted *versus *measured tumor cell fraction (C). In (A) and (C) each point represents the data of a single biopsy. In (A) the color code for tumor cell fractions (%) is shown. Spectral assignments are abbreviated in (B): β-Glc, β-glucose; Lac, lactate; *m*-Ino, *myo*-inositol; Cre, creatine; Tau, taurine; GPC, glycerophosphocholine; PC, phosphocholine; Cho, choline. The Pearson correlation coefficient and *p*-value are marked in (C). Total residual y-variance and root mean square error of prediction were minimised by retaining 2 PCs in the model. These two PCs accounted for 45% of the total x-variation, and 66% of the total y-variation.

In contrast to the apoptotic cell density, the tumor cell fraction showed a strong association to the spin-echo spectral profile (Figure 4), whereas the relationship to the standard pulse-acquire spectra was weaker (data not shown). Two principal components were retained in the model, and samples with high tumor cell fraction had high score values for the first principal component (PC1) compared to those with low tumor cell fraction (Figure 4A). The former samples were associated with increased levels of lactate, creatine, GPC, and PC and lower levels of glucose, *myo*-inositol, taurine, and choline, as could be seen from the loading profile of PC1 (Figure 4B). There was a highly significant correlation between the predicted and measured tumor cell fraction (*r *= 0.81, *p *< 0.001) (Figure 4C).

**Figure 5:**
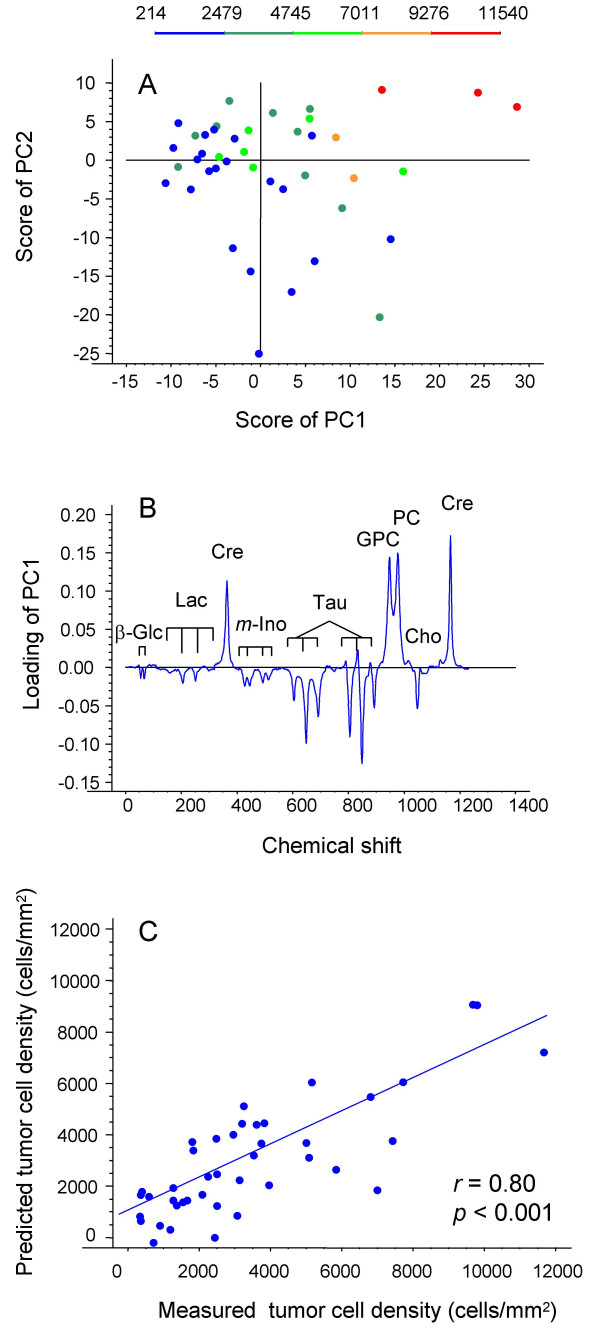
Score plot of first principal component (PC1) *versus *second principal component (PC2) from partial least square (PLS) regression analysis of tumor cell density to spin-echo spectral data (A), the corresponding loading profile of PC1 (B), and the predicted *versus *measured tumor cell density (C). In (A) and (C) each point represents the data of a single biopsy. In (A) the color code for tumor cell densities (cells/mm^2^) is shown. Spectral assignments are abbreviated in (B): β-Glc, β-glucose; Lac, lactate; *m*-Ino, *myo*-inositol; Cre, creatine; Tau, taurine; GPC, glycerophosphocholine; PC, phosphocholine; Cho, choline. The Pearson correlation coefficient and *p*-value are marked in (C). Total residual y-variance and root mean square error of prediction were minimised by retaining 3 PCs in the model. These three PCs accounted for 52% of the total x-variation, and 65% of the total y-variation.

PLS analysis of tumor cell density showed similar results as for tumor cell fraction, a strong association to the spin-echo spectral profile (Figure 5), whereas the relationship to the standard spectra was weaker (data not shown). The model consisted of three principal components, and samples with high tumor cell density had high score of PC1 compared to the other samples (Figure 5A). The metabolites associated with a high PCI score and therefore a high tumor cell density were the same as those associated with a high tumor cell fraction, although high levels of GPC and PC and low levels of choline seemed to be more dominant and high levels of lactate were less important (Figure 5B). The correlation between the predicted and measured tumor cell density was also comparable to that achieved for tumor cell fraction (*r *= 0.80, *p *< 0.001) (Figure 5C).

### Relationships between histopathology, metabolite concentrations, and metabolite ratios

Analysis of individual metabolites and metabolite ratios against the histopathological parameters led to lower correlation coefficients than found in the PLS analysis, however, significant correlations were found (Table 1). Apoptotic cell density was the only one showing a significant relationship to the ratio of fatty acid -CH_2 _to -CH_3 _(*r *= 0.36; *p *= 0.018). Hence, apoptotic samples had a high content of fatty acid - CH_2 _compared to fatty acid - CH_3_, regardless of tumor cell fraction and tumor cell density. Apoptotic cell density was not related to any of the other metabolites, in agreement with the PLS result showing that the lipid region was most important. Tumor cell density showed a positive correlation to GPC concentration (*r *= 0.35; *p *= 0.024) and an even stronger relationship to the ratio of GPC to choline (*r *= 0.53; *p *< 0.001), whereas tumor cell fraction was inversely correlated to glucose concentration (*r *= -0.52; *p *= 0.001), consistent with the PLS results. Of notice is that the necrotic sample had the third highest fatty acid -CH_2 _to -CH_3 _ratio, and therefore a characteristic similar to the highly apoptotic samples. Moreover, GPC concentration and ratio of GPC to choline were relatively low in this sample, resembling samples with low tumor cell density.

## Discussion

Detailed characterization of intact samples from cervical carcinomas by use of HR MAS MRS enabled identification of metabolites associated with apoptotic cell density, tumor cell fraction, and tumor cell density in our study. We showed that the samples were well preserved and that no apoptosis or cell death was induced during spectrum acquisition, in accordance with recent studies showing stable expression of apoptosis promoting genes during the experiment [18]. These findings justified direct correlation studies between MR data and histopathology and exploration of the underlying causes of the spectral profiles. Metabolites that were characteristic of apoptosis and differed from those associated with tumor cell fraction and tumor cell density were identified. The strongest relationship to the histopathology was achieved by including the spectral profiles of several metabolites in the analyses, showing that the combined metabolic information was important.

Apoptotic cell density correlated with the standard pulse-acquire spectra but not with the spin-echo profiles, suggesting that the lipid region contained the major information on apoptosis. In agreement with this hypothesis, the ratio of fatty acid - CH_2 _to - CH_3_, but none of the choline-containing metabolites, creatine, taurine, glucose, or lactate, was associated with apoptosis when individual metabolites and metabolite ratios were considered. Similar relationships between fatty acid -CH_2 _to -CH_3 _and apoptosis have also been reported in MR spectroscopy studies on Jurkat T-cell cultures treated with doxorubicin [8]. A weaker correlation was, however, found in our study probably because of the smaller range of apoptotic cell densities. The elevated -CH_2 _to -CH_3 _ratio in highly apoptotic tissues indicates increased fatty acid -CH_2 _chain length and/or increased degree of fatty acid saturation as apoptosis develops. The cause of this observation is not known, both dynamic and/or compositional changes in the plasma membrane and in cytoplasmic lipid droplets have been proposed [6]. The fatty acid - CH_2 _to -CH_3 _ratio was not significantly influenced by tumor cell fraction or tumor cell density in our study, but reflected the apoptotic activity *per se*, suggesting that this ratio can be used as an independent measure of apoptosis in cervical carcinomas.

Spectral characteristics associated with tumor cell fraction and tumor cell density were found in the region above 3 ppm. Choline, PC, and GPC were major metabolites contributing to the correlation in both cases, as verified from the loading profiles in the PLS analysis. High levels of GPC and PC and low levels of choline indicate activation of the phosphatidylcholine pathway and consequently a high membrane turnover and/or activation of cell proliferation and survival signalling [24,25], consistent with a high tumor cell fraction or tumor cell density. Up-regulation of the choline transport through the activation of choline kinase, leading to the observed changes in the choline metabolites, have been reported for many tumor types and is thought to be a common feature of cancers [26]. GPC concentration and GPC to choline ratio correlated with the tumor cell density in analysis of individual metabolites and ratios, whereas tumor cell fraction showed no such correlation, probably reflecting the cellular rather than extracellular localization of these metabolites and therefore a major dependence on the number of cells in the sample. Choline-containing metabolites have previously been shown to distinguish carcinomas and liposarcomas from non-cancerous tissue in HR MAS spectra [11-16]. Our results suggest a quantitative utilization of these data to assess tumor cell density in cervical cancer samples.

Creatine, taurine, glucose, and lactate also contributed to the strong correlation between the spectral profiles and tumor cell fraction and tumor cell density. Increased levels of creatine and taurine have been observed in HR MAS spectra of breast cancers as compared to non-malignant tissue [14], consistent with our results. The mechanisms underlying these findings have not been clarified, since taurine appears to have different functions in different tissues [27]. In contrast, the glucose and lactate data could be explained from nutrient depletion and increased anaerobe metabolism in cervical tumors as compared to normal tissues [28,29]. The MR signals of these metabolites originate from both the extracellular and cellular compartments [29], probably explaining the stronger relationship to tumor cell fraction than to tumor cell density. We have previously reported low glucose level in HR MAS spectra of cervical carcinomas as compared to normal samples [13]. Our present results indicate that this level can be a quantitative marker of tumor cell fraction in the carcinoma samples.

It was assumed that the spectral changes in lipid metabolites were caused solely by apoptosis, and not necrosis, in our work. Previous studies have shown that necrosis also may lead to changes in these metabolites [30]. It cannot be excluded that minor extent of necrosis was present in the samples, although not detected in analyses, and influenced the results. The only necrotic sample identified had spectral characteristics that resembled the highly apoptotic tissues with respect to lipid metabolites and tissues with low tumor cell density with respect to GPC concentration and ratio of GPC to choline. Further investigations, including both necrotic and apoptotic tissues, are therefore needed to clarify whether the two modes of cell death can be distinguished in the spectra or if the elevated fatty acid -CH_2 _to -CH_3 _ratio can be used as a measure of cell death in general.

## Conclusion

Our studies showed that the apoptotic activity of cervical carcinomas was associated with the lipid metabolites in HR MAS MR spectra, whereas tumor cell fraction and tumor cell density were related to cholines, creatine, taurine, glucose, and lactate. This suggests that HR MAS MRS may be used to explore the role of apoptosis in the course of the malignant disease and to achieve increased insight into the metabolic changes that occur during growth and apoptosis development. The spectral information specific of tumor load and tumor cell density allows for a more complete picture of the sample histopathology that is needed for a proper interpretation of the apoptosis data. Our results also indicate that HR MAS MRS may be utilized for monitoring of the therapeutic response of cervical carcinomas through the detection of treatment induced cell death. The detailed information on changes in apoptosis, tumor cell fraction, and tumor cell density that can be achieved by metabolic mapping of biopsies taken at different time points throughout the treatment would be useful in judgement of the therapeutic effect.

## Competing interests

The author(s) declare that they have no competing interests.

## Authors' contributions

HL conceived and designed the study, analysed histological sections, and wrote the article. BS, TFB, and LRJ performed the HR MAS MR experiments, analysed the spectral data against histopathology and helped to draft the manuscript. GBK and KS contributed with clinical samples and discussions. IG contributed to the conception and design of the study, the data analyses, and writing the article. All authors read and approved the final manuscript.

## Pre-publication history

The pre-publication history for this paper can be accessed here:



## Figures and Tables

**Table 1:** Correlations between HR MAS MR metabolites and histopathology of cervical carcinomas

	**Apoptotic cell density**		**Tumor cell fraction**		**Tumor cell density**	
	***r***^**1**^	***p***^**1**^	***r***	***p***	***r***	***p***
Concentrations^2^						
β-Glc		NS	-0.52	0.001		NS
GPC		NS		NS	0.35	0.024
Ratios						
GPC/Cho		NS		NS	0.53	< 0.001
FA-CH_2_/FA-CH_3_	0.36	0.018		NS		NS

^1^Correlation coefficients and *p*-values from Pearson correlation analysis. A threshold value of p = 0.05 was used for significance; NS means not significant.

^2^Spectral assignments are abbreviated β-Glc, β-glucose; GPC, glycerophosphocholine; Cho, choline; FA, fatty acid.
